# Upfront allogeneic transplantation versus JAK inhibitor therapy for patients with myelofibrosis: a North American collaborative study

**DOI:** 10.1038/s41409-023-02146-6

**Published:** 2023-11-08

**Authors:** Dawn Maze, Murat O. Arcasoy, Ryan Henrie, Sonia Cerquozzi, Rammurti Kamble, Samer Al-Hadidi, Abdulraheem Yacoub, Anurag K. Singh, Mahmoud Elsawy, Shireen Sirhan, Elliot Smith, Curtis Marcoux, Auro Viswabandya, Andrew Daly, Hassan Sibai, Caroline McNamara, Yuliang Shi, Wei Xu, Katherine Lajkosz, Lynda Foltz, Vikas Gupta

**Affiliations:** 1 The Princess Margaret Cancer Centre, University of Toronto, Toronto, ON Canada; 2 Duke Cancer Institute, Duke University School of Medicine, Durham, NC USA; 3 Division of Hematology, St. Paul’s Hospital, University of British Columbia, Vancouver, BC Canada; 4 Tom Baker Cancer Centre, Alberta Health Service Calgary Zone, University of Calgary, Calgary, AB Canada; 5 Center for Cell and Gene Therapy, Baylor College of Medicine and Houston Methodist Hospital, Houston, TX USA; 6 Myeloma Section, Winthrop P. Rockefeller Cancer Institute, University of Arkansas for Medical Sciences, Little Rock, AR USA; 7 Division of Hematology and Oncology, University of Kansas Medical Center, Kansas City, KS USA; 8 Division of Hematology, Dalhousie University, Halifax, NS Canada; 9 Jewish General Hospital, McGill University, Montreal, QC Canada; 10 Department of Biostatistics, Princess Margaret Cancer Centre, University Health Network, Toronto, ON Canada; 11 Department of Statistics and Actuarial Science, University of Waterloo, Waterloo, ON Canada; 12 Dalla Lana School of Public Health, University of Toronto, Toronto, ON Canada

**Keywords:** Myeloproliferative disease, Stem-cell therapies

## Abstract

Allogeneic hematopoietic cell transplantation (HCT) is the only curative therapy for myelofibrosis (MF) and is recommended for patients with higher risk disease. However, there is a risk of early mortality, and optimal timing is unknown. JAK inhibitor (JAKi) therapy may offer durable improvement in symptoms, splenomegaly and quality of life. The aim of this multicentre, retrospective observational study was to compare outcomes of patients aged 70 years or below with MF in chronic phase who received upfront JAKi therapy vs. upfront HCT in dynamic international prognostic scoring system (DIPSS)-stratified categories. For the whole study cohort, median overall survival (OS) was longer for patients who received a JAKi vs. upfront HCT, 69 (95% CI 57–89) vs. 42 (95% CI 20–not reached, NR) months, respectively (*p* = 0.01). In patients with intermediate-2 and high-risk disease, median OS was 55 (95% CI 36–73) months with JAKi vs. 36 (95% CI 20–NR) months for HCT (*p* = 0.27). An upfront HCT strategy was associated with early mortality and difference in median OS was not observed in any risk group by 5 years of follow-up. Within the limitations of a retrospective observational study, we did not observe any benefit of a universal upfront HCT approach for higher-risk MF.

## Introduction

Myelofibrosis (MF), which may occur de novo as primary MF (PMF) or following essential thrombocythemia (PET-MF) or polycythemia vera (PPV-MF), is a chronic myeloproliferative neoplasm characterized by constitutional symptoms, hepatosplenomegaly, cytopenias, an increased risk of vascular complications, and a risk of transformation to acute leukemia. The clinical features and prognosis of MF are variable; some patients may remain relatively asymptomatic with stable disease for many years, while others suffer a more aggressive clinical course with debilitating symptoms and/or early transformation to accelerated- or blast-phase (AP/BP) disease. Prognostic models such as the Dynamic International Prognostic Scoring System (DIPSS) [1] and DIPSS-Plus [2], as well as models incorporating molecular genetic features, such as MIPSS-70 [3], MIPSS-70 version 2.0 [4], and the personalized risk calculator by Grinfeld et al. [5], play an important role in risk stratification to identify those patients who are predicted to be at higher risk for early transformation to acute leukemia and poorer overall survival.

Conventional treatments such as hydroxyurea, glucocorticoids, androgens, and most recently, JAK inhibitor (JAKi) therapy, facilitate symptom control, spleen size reduction, and control of myeloproliferation, however, they have no clear disease-modifying effect. HCT is the only curative option for patients with MF and current expert consensus is to offer HCT to patients with DIPSS intermediate (int)-2 and high-risk disease and those with int-1 risk disease and additional risk factors [6]. However, high non-relapse mortality necessitates careful risk-benefit considerations for individual patients who may have good quality of life and relatively low risk of disease progression in the short term. On the other hand, the success of allogeneic transplant declines significantly once the disease has transformed into acute leukemia [7, 8], so it is imperative that eligible higher-risk patients are identified while the disease is still in the chronic phase. There are no published prospective studies comparing outcomes of HCT vs. non-HCT therapy for MF. Retrospective studies have demonstrated a long-term survival advantage of HCT for patients with higher risk DIPSS scores [9, 10], but the majority of the patients included in these studies were treated prior to the widespread use of JAKi therapy.

The introduction of JAKi in the past decade has altered the therapeutic landscape in MF. While JAKi has not been convincingly shown to modify the disease course or decrease the risk of leukemic transformation, a proportion of patients will derive durable clinical benefit from control of symptoms and splenomegaly [11, 12]. A common clinical dilemma is the optimal timing of HCT for a patient who is higher risk for disease progression, but responding well to JAKi and enjoying good quality of life. The goal of this study was to compare the outcomes of patients with MF who received upfront HCT to those treated with upfront JAKi therapy. Patients who proceeded directly to HCT or received a brief course of JAKi therapy as bridging to HCT were analyzed in the HCT group. Patients who received JAKi therapy with or without salvage HCT were analyzed in the JAKi group.

## Methods

### Patients

This multicentre study included adult patients up to 70 years of age with a diagnosis of MF who were initially seen at one of the eight participating centers in Canada and the United States and between January 1, 2012 and December 31, 2017 (research ethics board #18-5619). Ruxolitinib was approved for symptomatic MF by the FDA and Health Canada in 2011 and 2012, respectively, and was widely available after that time. Patients with a diagnosis of PMF, PET-MF or PPV-MF in chronic phase (blasts <10%) were included. Those with accelerated- or blast-phase MF were excluded. Patients were identified through local database searches and approval was obtained from the research ethics board at each participating center. The study was coordinated by the Elizabeth and Tony Comper MPN Program at the Princess Margaret Cancer Centre, Toronto.

### Definitions

To compare the planned, upfront treatment strategies, patients who received a short course of JAKi as a bridge to HCT (<6 months or documented plan of care) were analyzed in the upfront HCT group. Similarly, patients who were treated with JAKi, but received a HCT following JAKi failure (>12 months or documented plan of care) were analyzed in the JAKi group. Patients who received HCT between 6 and 12 months were analyzed according to the documented plan of care. The DIPSS score prior (within 6 months) to HCT or start of JAKi was determined for each group.

### Analysis

The primary outcome was overall survival (OS) in patients with DIPSS intermediate (int)-1 risk or higher who received upfront JAKi vs. HCT. To minimize selection and lead-time bias, OS was calculated from the start of JAK inhibitor and date of transplant, respectively. Group comparisons were performed using the Kruskal-Wallis test and Fisher’s exact test for continuous and categorical variables, respectively. OS estimates were calculated using the Kaplan-Meier method. Both crude OS estimates and adjusted OS estimates were visualized. The adjusted estimates incorporated diagnosis, age at initial visit, DIPSS, JAK2 type, cytogenetic abnormality, transfusion-requiring anemia and thrombocytopenia as covariates. Survival between groups was compared using the log-rank test. A multivariable Cox proportional hazards model comparing OS by treatment group while incorporating the aforementioned covariates was fit. The proportional hazards assumption was reviewed by inspecting the Schoenfeld residuals. The assumption was violated for the treatment effect, therefore, breakpoints in time were considered at 12, 18, and 24 months. The proportional hazards assumption held within each time period for only the 12 month breakpoint. Missing data was multiply imputed using ten iterations of the Multiple Imputation of Chained Equations, and results from the Cox proportional hazards models utilizing the imputed data were pooled using Rubin’s rules [13]. All statistical tests were two-sided, and *p* values less than 0.05 were considered statistically significant. Data analyses were performed using R version 4.2.1.

## Results

### Descriptive analysis

Between 2012 and 2017, 487 consecutive patients with chronic phase MF were seen at the study centers and 302 received JAKi or HCT as an initial treatment strategy. Of these, 171 (57%) had PMF and 131 (43%) had post-ET or post-PV MF. Driver mutations were identified as follows: *JAK2* in 200 patients (66%), *CALR* in 27 (9%), and *MPL* in 10 (3%). There were 11 patients (4%) classified as having triple-negative MF and the driver mutation status was unknown in 50 patients (17%). Next-generation sequencing results were available for 108 patients and of those, 61 (56%) were classified as high molecular risk (HMR), based on the presence of a pathogenic mutation in one or more of the following genes: *ASXL1*, *IDH1/2*, *SRSF2*, *EZH2*, *U2AF1* [4, 14]. Karyotype was available for 200 patients and 38 of those (19%) had a high-risk abnormality identified [2]. DIPSS score was int-1 in 126 patients (42%), int-2 in 150 (50%), and high in 26 (9%). Baseline characteristics are shown in Table 1. Regarding the patients who received HCT, 89 upfront and 50 upon JAKi failure, myeloablative conditioning regimen was used in 62 (45%) cases and reduced intensity in 70 (50%). This information was unavailable for seven patients (5%). A matched related donor was used in 48 (34%) patients, a matched unrelated donor in 64 (46%), a partially matched donor in 13 (9%), and a haploidentical donor in 8 (6%). Data were unavailable for the remaining six patients (4%).

**Table 1:** Demographic and clinical characteristics at initial visit of 302 patients with MF who received upfront JAKi or HCT.

Characteristic	Upfront JAKi^a^ *n* = 213	Upfront HCT *n* = 89	*P* value
Age			
- Median (range)	61 (16–70)	57 (28–69)	<0.01
Diagnosis, *n* (%)			
- PMF	117 (55)	54 (61)	0.14
- Post-ET MF	46 (22)	23 (26)	
- Post-PV MF	50 (23)	12 (13)	
Gender, *n* (%)			
- Female	79 (37)	33 (37)	1.00
- Male	134 (63)	56 (63)	
DIPSS, *n* (%)^b^			
- Intermediate-1	98 (46)	28 (31)	0.01
- Intermediate-2	94 (44)	56 (63)	
- High	21 (10)	5 (6)	
Transfusion-requiring anemia, *n* (%)			
- Yes	172 (81)	61 (69)	0.03
- No	41 (19)	28 (31)	
Thrombocytopenia, platelet count <100 × 10^9^/L			
- Yes	171 (80)	71 (80)	1.00
- No	42 (20)	18 (20)	
Driver mutation, *n* (%)			
- *JAK2*	147 (69)	53 (60)	0.42
- *CALR*	20 (9)	7 (8)	
- *MPL*	7 (3)	3 (3)	
- Triple negative	6 (3)	5 (6)	
- Two driver mutations	3 (1)	1 (1)	
- Unknown	30 (14)	20 (22)	
High molecular risk, *n* (%)^c^			
- Yes	40 (19)	21 (24)	0.61
- No	36 (17)	13 (15)	
- Unavailable	137 (64)	55 (62)	
Cytogenetics, *n* (%)			
- Normal	78 (37)	35 (39)	0.22
- Unfavorable^d^	22 (10)	16 (18)	
- Other abnormal	37 (17)	12 (13)	
- Unavailable	76 (36)	26 (29)	

*JAKi* JAK inhibitor therapy, *HCT* allogeneic hematopoietic cell transplantation, *MF* myelofibrosis, *PMF* primary myelofibrosis, *ET* essential thrombocythemia, *PV* polycythemia vera, *DIPSS* dynamic international prognostic scoring system.

^a^In the upfront JAKi group 50 patients underwent subsequent HCT for JAKi failure.

^b^At the time of initiation of JAKi or HCT.

^c^Pathogenic mutation in at least one of the following genes: *ASXL1, IDH1/2, SRSF2, EZH2, U2AF1* Q157.

^d^+8, −7/7q-, i(17q), inv(3), −5/5q, 12p-, or 11q23 rearrangements.

**Fig. 1:**
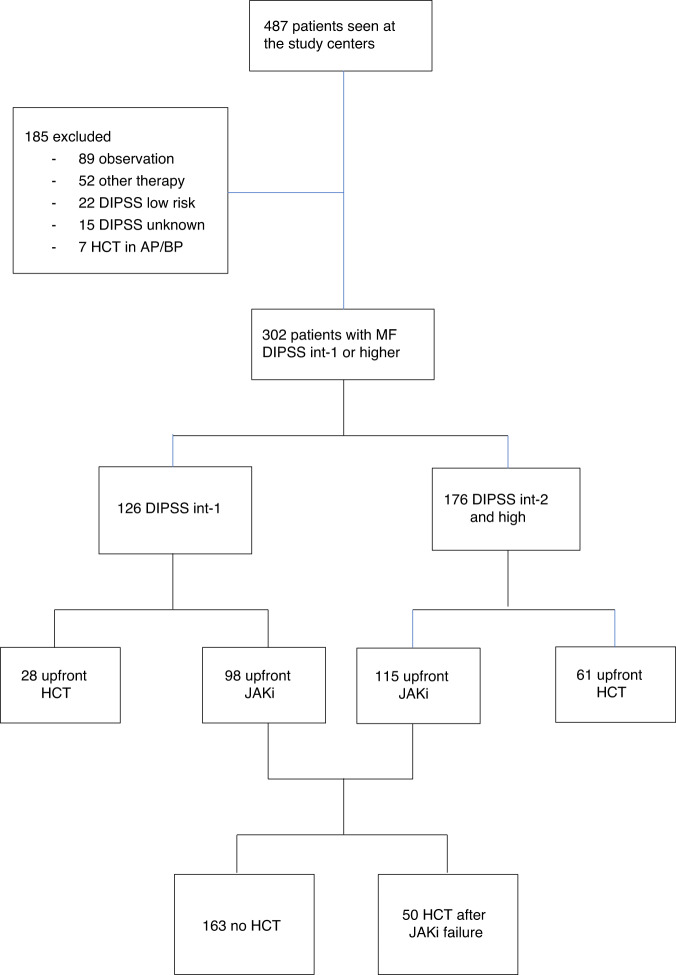
Flow diagram demonstrating treatment group assignment. MF myelofibrosis, DIPSS dynamic international prognostic scoring system, Int Intermediate, AP accelerated phase, BP blast phase, JAKi JAK inhibitor therapy, HCT allogeneic hematopoietic cell transplantation.

An upfront HCT strategy was used in 89 patients and an upfront JAKi strategy in 213 patients. Of the latter, 50 patients went on to receive HCT following JAKi failure (Fig. 1). Patients who received an upfront HCT strategy were younger, with a median age of 57 (range 28–69) years compared to 61 (16–70) for those treated with upfront JAKi (*p* < 0.01). The proportion of patients with a DIPSS score of int-2 was significantly higher in the HCT cohort, 63% vs. 44%, and the proportion of patients with a score of int-1 was significantly higher in the JAKi cohort, 46% vs. 31%, *p* = 0.01. More patients in the JAKi cohort had transfusion-requiring anemia, 81% vs. 69%, *p* = 0.03. Other baseline characteristics were similar between the groups. The median duration of follow-up was 49 (range 0–191) months.

### Survival outcomes

**Fig. 2:**
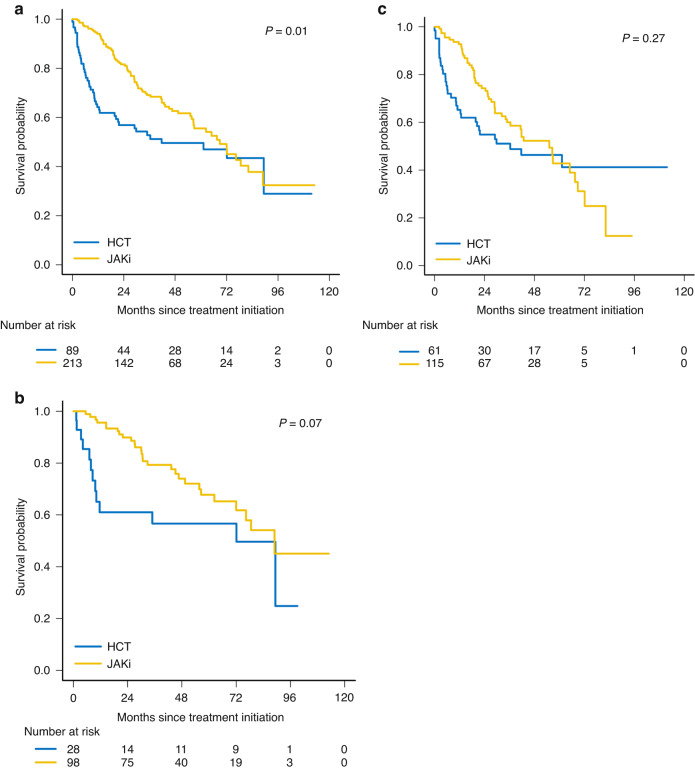
Kaplan-Meier survival curves for patients who received upfront JAKi vs. HCT. Yellow curves represent OS probability of patients who received upfront JAKi and blue curves represent that of patients who received upfront HCT for (**a**) the entire cohort of 302 patients with MF DIPSS int-1 or higher, (**b**) 126 patients with DIPSS int-1, and (**c**) 176 patients with DIPSS int-2 or high risk.

The median OS of the full cohort of MF patients with DIPSS int-1 or higher was longer in those who were managed with an upfront JAKi strategy: 69 (95% CI: 57–89) months for patients in the upfront JAKi group vs. 42 (95% CI: 20–not reached, NR) months in the upfront HCT group (*p* = 0.01). The JAKi arms had longer median OS when DIPSS risk groups were separated as well, but these differences were not statistically significant. For patients with int-1 risk disease the median OS was 79 (95% CI: 71–89%) months in the upfront JAKi group vs. 57 (95% CI: 40– 80%) in the upfront HCT group (*p* = 0.07). Given the small number of patients with high-risk DIPSS, these patients were combined with the int-2 cohort for analysis. The median OS of patients with int-2 and high-risk disease was 55 (95% CI: 36–72) months in the upfront JAKi group vs. 36 (95% CI: 20–NR) months in the upfront HCT group (*p* = 0.27; Fig. 2a–c).

At 36 months, survival was superior in the JAKi group for the full cohort (69%, 95% CI 63–76% vs. 53%, 95% CI 43–65% *p* = 0.01) and the int-1 cohort (79%, 95% CI 71–89% vs. 57%, 95% CI 40–80%, *p* = 0.04). At 60 months, no significant survival differences were observed in any of groups (Table 2). In the first 12 months, 43 patients died; 31 in the HCT group and 12 in the JAKi group. In the HCT group, 24 (77%) deaths were treatment-related, 2 (7%) were due to disease progression or relapse, and 5 (16%) were considered to be due to other causes, including cardiovascular events, bleeding, and other malignancies. In the JAKi group, 6 (50%) deaths were attributed each to disease progression and other events.

**Table 2:** Survival of patients with MF who received upfront HCT vs. JAKi in DIPSS-stratified categories.

	36-month survival, median (95% CI)			60-month survival, median (95% CI)		
	JAKi	HCT	*P* value	JAKi	HCT	*P* value
Total cohort (*n* = 302)	0.69 (0.63-0.76)	0.53 (0.43-0.65)	0.01	0.56 (0.48-0.65)	0.50 (0.48-0.65)	0.41
Int-1 (*n* = 126)	0.79 (0.71-0.89)	0.57 (0.40-0.80)	0.04	0.68 (0.57-0.80)	0.57 (0.40-0.80)	0.34
Int-2, High (*n* = 176)	0.60 (0.35-0.62)	0.51 (0.40-0.71)	0.28	0.43 (0.31-0.58)	0.46 (0.35-0.62)	1.00

*MF* myelofibrosis, *JAKi* JAK inhibitor therapy, *HCT* allogeneic hematopoietic cell transplantation, *PMF* primary myelofibrosis, *DIPSS* dynamic international prognostic scoring system, *CI* Confidence interval.

### Predictors of overall survival

After adjusting for other predictors, in the first 12 months OS was superior amongst patients treated with JAKi, HR = 0.14 (95% CI 0.07–0.28; *p* < 0.01). However, beyond 12 months OS was inferior amongst JAKi-treated patients, HR = 1.98 (95% CI 1.08–3.61; *p* = 0.03; Table 3). Higher DIPSS at the time of intervention (HR 1.63; 95% CI 1.07–2.48; *p* = 0.03 and 2.43; 95% CI 1.25–4.70; *p* = 0.01 for int-2 and high risk, respectively) and thrombocytopenia (HR 1.55; 95% CI 1.02–2.36; *p* = 0.04) were associated with worse OS. Diagnosis of post-ET/PV MF was associated with better OS compared to PMF (HR 0.65; 95% CI 0.45–0.94; *p* = 0.03). Age, JAK2 mutation, cytogenetic abnormality and transfusion-requiring anemia were not associated with overall survival.

**Table 3:** Multivariate Cox proportional hazard model for overall survival.

Variables	HR (95% CI)	*P* value
OS (≤12 months)		
- HCT	Ref	<0.01
- JAKi	0.14 (0.07–0.28)	
OS (>12 months)		
- HCT	Ref	0.03
- JAKi	1.98 (1.08–3.61)	
Diagnosis		
- PMF	Ref	<0.03
- Post-ET/PV MF	0.65 (0.45–0.94)	
Age at initial visit	1.02 (1.00–1.04)	0.25
DIPSS^a^		
- Intermediate-1	Ref	
- Intermediate-2	1.63 (1.07–2.48)	0.03
- High	2.43 (1.25–4.70)	0.01
Transfusion-requiring anemia		
- No	Ref	0.36
- Yes	1.23 (0.79–1.90)	
Thrombocytopenia, platelet count < 100 × 10^9^/L		
- No	Ref	0.04
- Yes	1.55 (1.02–2.36)	
JAK2		
- Wild type	Ref	0.88
- Mutated	0.97 (0.66–1.44)	
Unfavorable cytogenetic abnormality^b^		
- Absent	Ref	0.52
- Present	1.18 (0.72–1.93)	

*HR* Hazard ratio, *CI* Confidence interval, *Ref* Reference, *OS* Overall survival, *MF* myelofibrosis, *PMF* primary myelofibrosis, *ET* essential thrombocythemia, *PV* polycythemia vera, *DIPSS* dynamic international prognostic scoring system, *Int* intermediate.

^a^At the time of initiation of JAKi or HCT.

^b^+8, -7/7q-, i(17q), inv(3), -5/5q, 12p-, or 11q23 rearrangements.

### JAKi bridging prior to transplant

**Fig. 3:**
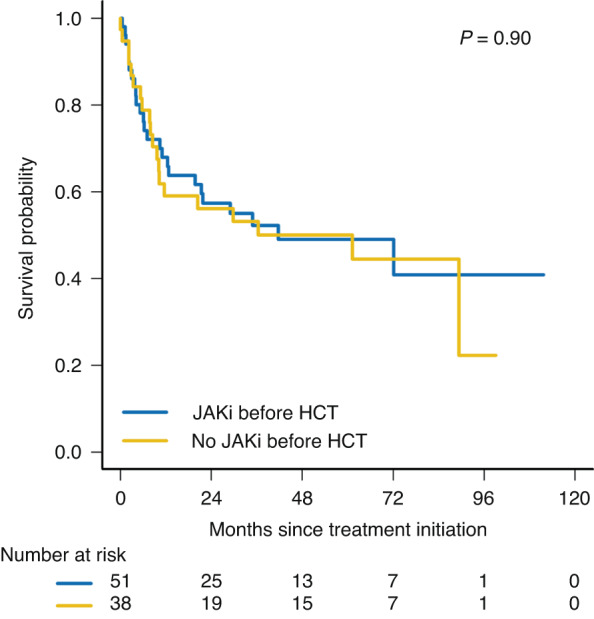
Kaplan-Meier survival curves for patients who received JAKi bridging vs. no bridging therapy prior to HCT. Eighty-nine patients received upfront HCT. The blue curve represents the OS probability of the 51 patients who received JAKi bridging therapy prior to HCT. The yellow curve represents that of patients who did not receive bridging therapy.

Of the 89 patients who received upfront HCT, 51 patients received JAKi as bridging therapy prior to the planned HCT and 36 patients did not. The survival at 60 months was 49% (95% CI: 36–66%) for patients who received JAKi bridging and 50% (95% CI: 36–70%) for those who proceeded directly to HCT without JAKi bridging (*p* = 0.93; Fig. 3).

## Discussion

In this multicentre study of adults up to age 70 years with chronic phase MF, the median survival of patients treated with a JAKi upfront was longer than that of patients treated with upfront HCT. At 36 months, survival was longer in the JAKi arm and, although the shape of the survival curve for the HCT arm suggests a plateau, no significant difference was detected by 60 months. Early morality was observed in the HCT arm. Higher DIPSS, thrombocytopenia, primary vs. secondary MF and, after 12 months, treatment on the JAKi arm, were associated with inferior survival.

Allogeneic transplantation is the only potentially curative treatment option for patients with MF. Early studies demonstrated the feasibility of engraftment and long-term disease control [15–18], and more recently, widespread adoption of reduced-intensity conditioning regimens has resulted in lower rates of non-relapse mortality [19]. HCT is currently recommended for eligible patients with DIPSS int-2 or high-risk MF and select patients with lower-risk disease with adverse cytogenetic or molecular genetic features [6]. This is on the basis of retrospective comparative studies that have demonstrated a survival advantage of HCT compared to non-HCT therapy in patients predicted to have poor survival based on DIPSS [9, 10, 20]. Notably, the majority of patients treated with non-HCT therapy in these prior studies did not receive a JAKi. It is possible that the relatively delayed benefit of HCT observed in this current study compared to others is related to the use of JAKi therapy in the non-HCT group.

JAKi therapy improves constitutional symptoms and splenomegaly and improves quality of life in patients with MF. However, its potential on survival continues to be debated [21]. Post hoc analysis of pooled data from the COMFORT [22, 23] studies suggested improved OS with ruxolitinib but these studies were not powered to evaluate survival [12, 24]. A subsequent review by the Cochrane Collaboration concluded that the quality of evidence was low and there is uncertainty as to whether the drug influences overall survival compared to placebo or best available therapy [25]. In a recent cohort study of 1010 patients with MF, median OS were significantly longer in patients treated with ruxolitinib compared to those who received hydroxyurea [26]. Similar results have been reported for the newer JAK inhibitors, fedratinib [27], and momelotinib [28], suggesting a potential survival benefit in responding patients.

While the role of transplant in higher-risk MF is established as a potentially curative therapy, its optimal timing remains unknown. This question is particularly relevant in patients who are responding well to JAKi therapy. Advances in supportive care, transplant conditioning, and graft-versus-host disease (GVHD) prophylaxis have improved outcomes, but morbidity and mortality following HCT remain substantial. A delayed transplant approach is associated with the patient being older at the time of transplant and increases the likelihood of comorbidities or a decline in performance status that might increase transplant-related complications or even preclude a transplant altogether. Certainly, transplant outcomes are inferior if the disease progresses to accelerated or blast phase [7, 8]. On the other hand, if performed too early, transplant may compromise quality and quantity of life. In a recent study from the CIBMTR, HCT was associated with survival benefit over non-HCT therapy in patients with MF with DIPSS int-1 risk and higher that was observed beyond 1 year of treatment arm assignment [10]. In an effort to further evaluate the optimal timing for HCT in MF, Cipkar et al. performed Markov modeling on a hypothetical patient cohort [29]. Their modeling supports an early HCT strategy for patients with higher risk MF, with gains in life expectancy peaking at 16.6 and 9.7 months for patients with DIPSS int-2 and high risk MF, respectively. The JAKi group in this study included patients who were treated with an upfront JAKi strategy, regardless of subsequent treatment. Subsequent therapy was transplant for 50 (23.5%) patients and other, non-transplant, therapies for 163 (76.5)%. Due to the retrospective and multicentre nature of this study, we were unable to capture the exact reasons for not proceeding to HCT. This study was not designed to, and cannot, address the question of early vs. delayed HCT strategies.

While most of the evidence supporting HCT for MF has been based on DIPSS, advances in the understanding of molecular pathogenesis and risk stratification will likely translate into more personalized decision-making in the near future. The MPN driver mutation impacts the disease course [30], as well as the presence of high-risk mutations, including *ASXL1*, *EZH2*, *IDH1/2*, *SRSF2,* and *U2AF1* Q157 [4, 14]. Mutational data has formed the basis of the genetically inspired prognostic scoring system for primary myelofibrosis (GIPSS) [31], is included along with clinical risk factors in the MIPSS70 and MIPSS70 plus v2.0 [3, 4], and may also help predict response to ruxolitinib [32, 33]. More recently, Grinfeld and colleagues used clinical and genomic variables to develop a personalized model for prediction of clinical outcomes in patients with MPN [5]. Molecular data has also been included, along with clinical and transplant-specific variables, into the myelofibrosis transplant scoring system (MTSS), which predicts outcomes post-HCT [34]. Using all of the available information, along with careful exploration of patients’ goals and preferences, will facilitate decision making around HCT and its appropriate timing.

This multicentre study included a large number of patients with MF treated at academic centers in the United States and Canada. Strengths include a uniformly treated non-HCT cohort and long follow up. An important limitation is the retrospective, non-randomized nature of the study. It is possible that patients with subjectively worse disease or risk factors not captured by DIPSS were selected for early HCT, favouring the JAKi group; or that less fit patients were selected for JAKi therapy, potentially favouring the HCT group. A lack of data on potentially important comorbid conditions may have contributed to selection bias. The transplant cohort in this study had a lower median age and higher proportion of higher-risk DIPSS. As well, information about the clinical response to JAKi and factors contributing to the decision to proceed with HCT are limited. It is also important to note that the study period predates the widespread availability of NGS and so information about somatic mutations was available for only a subset.

In this large, multicentre, study the median OS was longer for patients with MF who were treated with upfront JAKi therapy than those who received upfront HCT. An upfront HCT strategy was associated with early mortality and a difference in median OS was not observed in any risk group by 5 years of follow up in the upfront HCT arm. Within the limitations of the retrospective nature of this study, we did not observe a benefit of a universal upfront HCT approach in any DIPSS-stratified category in patients with MF aged 70 years or less. Advances in genomic-based prognostication may help further define groups who are unlikely to have durable benefit from JAKi therapy and should be considered for early transplantation. Concerted collaborative efforts between MPN physicians and the transplant community will be required to understand the comparative outcomes of transplant and non-transplant therapies to decide the optimal timing of transplantation.

## Data Availability

The data that support the findings of this study are available from the authors upon reasonable request.
