# Correction: Upfront allogeneic transplantation versus JAK inhibitor therapy for patients with myelofibrosis: a North American collaborative study

**DOI:** 10.1038/s41409-023-02194-y

**Published:** 2024-01-26

**Authors:** Dawn Maze, Murat O. Arcasoy, Ryan Henrie, Sonia Cerquozzi, Rammurti Kamble, Samer Al-Hadidi, Abdulraheem Yacoub, Anurag K. Singh, Mahmoud Elsawy, Shireen Sirhan, Elliot Smith, Curtis Marcoux, Auro Viswabandya, Andrew Daly, Hassan Sibai, Caroline McNamara, Yuliang Shi, Wei Xu, Katherine Lajkosz, Lynda Foltz, Vikas Gupta

**Affiliations:** 1 The Princess Margaret Cancer Centre, University of Toronto, Toronto, ON Canada; 2 Duke Cancer Institute, Duke University School of Medicine, Durham, NC USA; 3 Division of Hematology, St. Paul’s Hospital, University of British Columbia, Vancouver, BC Canada; 4 Tom Baker Cancer Centre, Alberta Health Service Calgary Zone, University of Calgary, Calgary, AB Canada; 5 Center for Cell and Gene Therapy, Baylor College of Medicine and Houston Methodist Hospital, Houston, TX USA; 6 Myeloma Section, Winthrop P. Rockefeller Cancer Institute, University of Arkansas for Medical Sciences, Little Rock, AR USA; 7 Division of Hematology and Oncology, University of Kansas Medical Center, Kansas City, KS USA; 8 Division of Hematology, Dalhousie University, Halifax, NS Canada; 9 Jewish General Hospital, McGill University, Montreal, QC Canada; 10 Department of Biostatistics, Princess Margaret Cancer Centre, University Health Network, Toronto, ON Canada; 11 Department of Statistics and Actuarial Science, University of Waterloo, Waterloo, ON Canada; 12 Dalla Lana School of Public Health, University of Toronto, Toronto, ON Canada

**Keywords:** Myeloproliferative disease, Stem-cell therapies

Correction to: *Bone Marrow Transplantation* 10.1038/s41409-023-02146-6, published online 08 November 2023

The article Upfront allogeneic transplantation versus JAK inhibitor therapyfor patients with myelofibrosis: a North American collaborative study, written by Dawn Maze, Murat O. Arcasoy, Ryan Henrie, Sonia Cerquozzi, Rammurti Kamble, Samer Al-Hadidi, Abdulraheem Yacoub, Anurag K. Singh, Mahmoud Elsawy, Shireen Sirhan, Elliot Smith, Curtis Marcoux, Auro Viswabandya, Andrew Daly, Hassan Sibai, Caroline McNamara, Yuliang Shi, Wei Xu, Katherine Lajkosz, Lynda Foltz and Vikas Gupta was originally published electronically on the publisher’s internet portal on 8 November 2023 without open access. With the author(s)’ decision to opt for Open Choice the copyright of the article changed on 6 December 2023 to © The Author(s) 2023 and the article is forthwith distributed under a Creative Commons Attribution 4.0 International License, which permits use, sharing, adaptation, distribution and reproduction in any medium or format, as long as you give appropriate credit to the original author(s) and the source, provide a link to the Creative Commons licence, and indicate if changes were made. The images or other third party material in this article are included in the article’s Creative Commons licence, unless indicated otherwise in a credit line to the material. If material is not included in the article’s Creative Commons licence and your intended use is not permitted by statutory regulation or exceeds the permitted use, you will need to obtain permission directly from the copyright holder. To view a copy of this licence, visit http://creativecommons.org/licenses/by/4.0.

